# GLP-1 Suppresses Feeding Behaviors and Modulates Neuronal Electrophysiological Properties in Multiple Brain Regions

**DOI:** 10.3389/fnmol.2021.793004

**Published:** 2021-12-17

**Authors:** Xin-Yi Chen, Lei Chen, Wu Yang, An-Mu Xie

**Affiliations:** ^1^Department of International Medicine, Affiliated Hospital of Qingdao University, Qingdao, China; ^2^Department of Neurology, Affiliated Hospital of Qingdao University, Qingdao, China; ^3^Department of Physiology and Pathophysiology, School of Basic Medicine, Qingdao University, Qingdao, China

**Keywords:** GLP-1, electrophysiological property, feeding behavior, spontaneous firing activity, synaptic transmission

## Abstract

The glucagon-like peptide-1 (GLP-1) plays important roles in the regulation of food intake and energy metabolism. Peripheral or central GLP-1 suppresses food intake and reduces body weight. The electrophysiological properties of neurons in the mammalian central nervous system reflect the neuronal excitability and the functional organization of the brain. Recent studies focus on elucidating GLP-1-induced suppression of feeding behaviors and modulation of neuronal electrophysiological properties in several brain regions. Here, we summarize that activation of GLP-1 receptor (GLP-1R) suppresses food intake and induces postsynaptic depolarization of membrane potential and/or presynaptic modulation of glutamatergic or GABAergic neurotransmission in brain nuclei located within the medulla oblongata, pons, mesencephalon, diencephalon, and telencephalon. This review may provide a background to guide future research about the cellular mechanisms of GLP-1-induced feeding inhibition.

## Introduction

The pre-proglucagon (Gcg) gene product peptides include glucagon-like peptide 1 (GLP-1), GLP-2, oxyntomodulin (OXM), intervening peptide 1 (IP1), and glicentin. The GLP-1-producing preproglucagon (PPG) neurons located in the nucleus tractus solitarius (NTS) and the intermediate reticular nucleus of the medulla oblongata are the major source of endogenous GLP-1 in the central nervous system, which project widely throughout the central nervous system especially the autonomic control areas (Merchenthaler et al., 1999; Barrera et al., 2011; Llewellyn-Smith et al., 2011; Holt et al., 2019; Muller et al., 2019). Ablation of the PPG neurons in the NTS largely reduces the level of GLP-1 in the hypothalamus, brainstem, and spinal cord (Holt et al., 2019). In addition to the central source, peripheral GLP-1 is released from enteroendocrine L-cells in intestinal mucosa (Eissele et al., 1992) which plays an important role in regulating glucose homeostasis (Edwards et al., 1999; Williams, 2009). Furthermore, a small population of PPG neurons has been identified within the olfactory bulb with only local projection (Thiebaud et al., 2016). Central GLP-1 binds to GLP-1 receptor (GLP-1R) to exert many important effects including modulation of energy balance, cardiovascular system, learning and memory, rewarding effect of food, and thermogenesis (Trapp and Cork, 2015). GLP-1R belongs to G protein-coupled receptors with predominate Gα_s_ coupling, leading to activation of adenylate cyclase and in turn increased levels of cAMP (Mayo et al., 2003). GLP-1R expressing cells are widely expressed in mouse and non-human primate brain (Cork et al., 2015; Heppner et al., 2015). Recent immunocytochemistry revealed the distribution and subcellular localization of GLP-1R in rat brain (Farkas et al., 2021).

GLP-1 is involved in the regulation of food intake and energy metabolism. Both human clinical trials and animal experiments demonstrated that peripheral or central GLP-1 and GLP-1 analogs suppress food intake and reduce body weight (Turton et al., 1996; Hayes et al., 2008, 2011; Dossat et al., 2011; Heppner and Perez-Tilve, 2015). A recent study revealed that central and peripheral GLP-1 inhibits feeding behaviors through independent gut-brain circuits (Brierley et al., 2021). Activation of GLP-1R in a variety of brain regions, including the hypothalamus (Schick et al., 2003), mesolimbic system (Dossat et al., 2011; Alhadeff et al., 2012; Dickson et al., 2012), and hindbrain (Hayes et al., 2011; Alhadeff et al., 2014), reduces food intake. Drugs targeting GLP-1R have been used as weight loss and anti-diabetic glucose-lowering therapies (Heppner and Perez-Tilve, 2015).

The brain is the most intricate network structure which facilitates a concerted communication between single neurons, different neuronal populations, and remote brain (Gupta et al., 2020). Neurons are the basic structural and functional units in the central nervous system. The electrophysiological properties of neurons such as the spontaneous firing activities and the synaptic neurotransmission in the mammalian central nervous system reflect the neuronal excitability and the functional organization of the brain (Llinás, 1989, 2014). To date, measuring the electrophysiological features of neurons remains one of the most valuable methods to study the functional phenomena of the nervous system. The specific deficits of the electrophysiological properties contribute to some brain diseases (Bernard and Shevell, 2008; Klassen et al., 2011; Tai et al., 2014). Therefore, manipulation of the electrophysiological properties including the spontaneous firing activity of central neurons may play roles in the manifestation of some neurological disorders. For example, the electrophysiological characteristics of dopaminergic neurons in the substantia nigra pars compacta change before the appearance of motor symptoms in parkinsonian mice (Qi et al., 2017), while excitatory stimulation of dopaminergic neurons may improve the survival of the neurons (Michel et al., 2013). Many studies have demonstrated that GLP-1 suppresses feeding behaviors and modulates the spontaneous firing activities and/or glutamatergic or GABAergic neurotransmission in multiple brain regions. This review highlights the activation of GLP-1R-induced suppression of feeding as well as the modulation of neuronal electrophysiological properties of several brain regions in medulla oblongata, pons, mesencephalon, diencephalon, and telencephalon.

## Medulla Oblongata and Pons

The medullar oblongata in rodents and monkeys expresses a high level of GLP-1R (Merchenthaler et al., 1999; Cork et al., 2015; Heppner et al., 2015; Farkas et al., 2021). In human brain tissue of autopsies, GLP-1R is also expressed in the medullar oblongata including the area postrema, the dorsal motor nucleus of the vagus, and the NTS (Farr et al., 2016). GLP-1 modulates feeding behaviors in the medullar oblongata. Recently, Gaykema et al. (2017) reported that selectively chemogenetic stimulation of caudal medulla pre-proglucagon-producing neurons reduces food intake in both fed and fasted states and suppresses glucose production. Patch-clamp electrophysiological recordings in brain slices further demonstrated that chemogenetic activation selectively depolarizes neuronal membrane potential and increases the firing frequency of labeled medulla pre-proglucagon-producing neurons without affecting unlabeled neurons.

The NTS is the main source of endogenous GLP-1 within the brain (Barrera et al., 2011; Holt et al., 2019). Application of the stable GLP-1R analog exendin-4 into the medial subnucleus of the NTS (mNTS) reduces high-fat diet intake (Alhadeff and Grill, 2014; Table 1). However, electrophysiological studies revealed that GLP-1 or exendin-4 does not change the spontaneous firing activity as well as the synaptic transmission suggesting lack of functional GLP-1R in PPG neurons (Hisadome et al., 2010). Consistent with the electrophysiological results, the morphological study showed a weak/faint expression of GLP-1R in the NTS. It is reported that astrocytes in NTS are components of the GLP-1 signaling system which is involved in food intake control (Reiner et al., 2016). Intracerebroventricular application of GLP-1R agonist binds to GLP-1R on both neurons and astrocytes in the NTS. Activation of GLP-1R induces an increase in intracellular Ca^2+^ in 40% of NTS astrocytes, while selective inhibition of astrocyte function in NTS abolishes exendin-4-induced inhibition of food intake (Reiner et al., 2016). Therefore, complex mechanisms in both neurons and astrocytes may be involved in GLP-1-induced modulation of food intake in the NTS.

**TABLE 1:** Activation of GLP-1R suppresses feeding behaviors and modulates neuronal electrophysiological properties in several brain nuclei.

Brain regions	Neurons	Associated effects in feeding behaviors		Electrophysiological effects of activating GLP-1R	GLP-1R agonists	References
		Activation of GLP-1R	Ablation of GLP-1R			
mNTS	PPG neurons	Reduction of high-fat diet intake	N/A	No change in firing activity and synaptic transmission	Exendin-4 GLP-1	Hisadome et al., 2010; Alhadeff and Grill, 2014

PBN	Unidentified neurons	Reduction of food intake and body weight	N/A	Increase in firing rate	Exendin-4	Richard et al., 2014

VTA	DAergic VTA-to-NAc projection neurons	Suppression of high-fat food intake	N/A	Increase of sEPSCs frequency Inhibition of mEPSCs	Exendin-4	Mietlicki-Baase et al., 2013; Wang et al., 2015

ARC	POMC neurons	N/A	N/A	Depolarization and increase in firing rate *via* TRPC5 channels Increase of EPSCs frequency	Liraglutide	Secher et al., 2014; He et al., 2019
	NPY/AgRP neurons	N/A	N/A	Hyperpolarization *via* enhanced GABA_A_ receptor-mediated neurotransmission	Liraglutide	Secher et al., 2014; He et al., 2019
	Kisspeptin (Kiss1)-expressing neurons	N/A	N/A	Depolarization and increase in firing rate	Liraglutide	Heppner et al., 2017

PVN	Unidentified neurons	Reduction of food intake	Increase of food intake and induction of obesity	Hyperpolarization *via* enhancement of inhibitory postsynaptic transmission Depolarization or inward current accompanied by an increase in membrane conductance	Exendin-4 GLP-1	Larsen et al., 1997; McMahon and Wellman, 1998; Acuna-Goycolea and van den Pol, 2004; Cork et al., 2015
	CRH neurons	N/A	N/A	Enhancement of EPSC amplitude		Liu et al., 2017

LH	Orexinergic neurons	N/A	N/A	Depolarization and increase in firing rate postsynaptically *via* sodium-dependent non-specific cationic conductance Enhancement of both glutamatergic and GABAergic neurotransmission presynaptically	Exendin-4	Acuna-Goycolea and van den Pol, 2004

PVT	Unidentified neurons	Reduction of food intake Decrease of food-seeking and food-motivated behaviors	N/A	Decrease in firing rate probably *via* suppression of glutamatergic synaptic transmission	Exendin-4	Ong et al., 2017

NAc	MSNs	Suppression of food intake	N/A	Reduction of evoked action potential postsynaptically Increase of mEPSCs frequency presynaptically	Exendin-4	Dossat et al., 2011; Mietlicki-Baase et al., 2014

BNST	Unidentified neurons	Food suppression during the dark phase	N/A	Inward current and depolarization accompanied by an increase in membrane conductance Increase or decrease in firing rate Hyperpolarization probably *via* opening of potassium channels	GLP-1	Cork et al., 2015 Williams et al., 2018

HC	CA1 neurons	Reduction of food intake and body weight	Increase of food motivated behaviors	Increase and then decrease in firing activity	Active fragment of GLP-1, GLP-1 (7-36) amide GLP-1	Oka et al., 1999; Hsu et al., 2015, 2018
			Depolarization in most hippocampal neurons, and hyperpolarization in a few neurons		Cork et al., 2015; Gullo et al., 2017	

OB	MCs	N/A	N/A	Increase of the excitability probably *via* inhibition of voltage-dependent potassium channel	GLP-1 Exendin-4	Thiebaud et al., 2016; Schwartz et al., 2021

*ARC, arcuate nucleus; BNST, bed nucleus of the stria terminalis; CRH, corticotropin-releasing hormone; EPSCs, excitatory postsynaptic currents; HC, hippocampus; LH, lateral hypothalamus; MCs, mitral cells; mEPSCs, miniature excitatory postsynaptic currents; mNTS, medial subnucleus of the nucleus tractus solitaries; MSNs, medium spiny neurons; N/A, not applicable; NAc, nucleus accumbens; NPY/AgRP, Neuropeptide Y/Agouti gene related peptide; OB, olfactory bulb; PBN, parabrachial nucleus; POMC, proopiomelanocortin; PVN, paraventricular nucleus; PVT, paraventricular thalamic nucleus; VTA, ventral tegmental area.*

**FIGURE 1:**
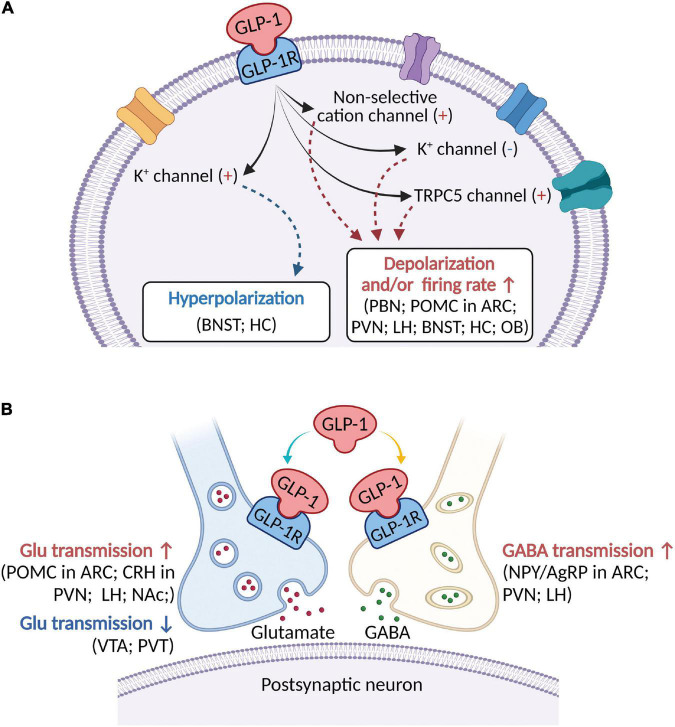
A schematic diagram describing the major electrophysiological effects of activating GLP-1R in brain areas involved in modulation of feeding behaviors. **(A)** GLP-1 (including its agonists) binds to postsynaptic GLP-1R to depolarize membrane potential and/or increase firing rate in most brain regions, but hyperpolarize membrane potential in a few brain areas. Several ionic mechanisms, including non-selective cation channel, K^+^ channel, and TRPC5 channel, may be involved in activation of GLP-1R-induced depolarization or hyperpolarization. **(B)** In addition to postsynaptic receptors, GLP-1 acts on presynaptic GLP-1R to modulate both glutamatergic and GABAergic neurotransmission. ARC, arcuate nucleus; BNST, bed nucleus of the stria terminalis; Glu, glutamate; CRH, corticotropin-releasing hormone; HC, hippocampus; LH, lateral hypothalamus; NAc, nucleus accumbens; NPY/AgRP, Neuropeptide Y/Agouti gene-related peptide; OB, olfactory bulb; PBN, parabrachial nucleus; POMC, proopiomelanocortin; PVN, paraventricular nucleus; PVT, paraventricular thalamic nucleus; VTA, ventral tegmental area.

The parabrachial nucleus (PBN) in the pons is associated with the regulation of feeding behaviors. The PBN receives direct GLP-1 projections from NTS neurons (Richard et al., 2014). Stimulation of GLP-1R with exendin-4 in the PBN reduces food intake and therefore decreases body weight in rats. Electrophysiological evidence further revealed that application of exendin-4 results in a remarkable increase in the spontaneous firing rate of the PBN neurons (Richard et al., 2014; Figure 1A). Using the methods of immuno-electron microscopy, Farkas et al. (2021) recently revealed a very widespread distribution of GLP-1R fibers in rat brain suggesting the possible presynaptic effects of GLP-1R in the central nervous system. As the external part of the lateral parabrachial nucleus (LPBN) expresses the highest density of GLP-1R immunoreactive fibers (Farkas et al., 2021), further electrophysiological studies are needed to study the possible presynaptic modulation of the electrophysiological activities of the PBN neurons.

## Mesencephalon

The ventral tegmental area (VTA) is a possible brain region for GLP-1-induced suppression of food intake. Functional study revealed that application of GLP-1R antagonist into the VTA attenuates peripheral application of exendin-4-induced anorectic effects (Mietlicki-Baase et al., 2013). Electrophysiological recordings revealed that exendin-4 increases the frequency of spontaneous excitatory postsynaptic currents (sEPSCs) of VTA dopaminergic neurons suggesting the possible presynaptic modulation of GLP-1R on glutamatergic terminals. Behavioral study also demonstrated that modulating AMPA/kainite, but not NMDA, receptor-mediated glutamatergic neurotransmission within VTA is involved in GLP-1-induced intake-suppressive effects (Mietlicki-Baase et al., 2013). In addition, intra-VTA application of exendin-4 suppresses high-fat food intake, which is consistent with the results of chemogenetic activation of endogenously released GLP-1 nerve terminals in the VTA (Wang et al., 2015). In contrast to the enhancement of spontaneous excitatory postsynaptic transmission (Mietlicki-Baase et al., 2013), using retrograde labeling of VTA to nucleus accumbens (NAc) medial shell projecting neurons, *in vitro* patch-clamp recordings showed that exendin-4 selectively inhibits the miniature excitatory postsynaptic currents (mEPSCs) within the dopaminergic VTA-to-NAc projection neurons (Wang et al., 2015; Figure 1B) suggesting the presynaptic inhibition of glutamatergic neurotransmission. As NAc is also an important brain region associated with GLP-1-induced feeding suppression, further electrophysiological studies are necessary to explore the contribution of glutamatergic neurotransmission to endogenously released GLP-1-induced suppression of high-fat food intake in the VTA.

## Diencephalon

The arcuate nucleus (ARC) of the hypothalamus plays a particularly important role in the central regulation of food intake (Bouret et al., 2004). Two distinct types of neurons within the ARC, proopiomelanocortin (POMC) and Neuropeptide Y (NPY)/Agouti gene-related peptide (AgRP) neurons, play important roles in energy balance and glucose homeostasis (Gautron et al., 2015; Caron et al., 2018). Activation of both the NPY/AgRP neurons and POMC neurons coordinates the activity of the paraventricular nucleus (PVN), promoting stimulation or inhibition of feeding, respectively. It is well known that the anti-diabetic drug, long-acting GLP-1R agonist, liraglutide reduces body weight. The highest level of GLP-1R expressing cells, detected by transgene expression (Cork et al., 2015), *in situ* hybridization (Merchenthaler et al., 1999; Heppner et al., 2015), and immunocytochemistry (Farkas et al., 2021), is present in the ARC. *In vitro* patch-clamp electrophysiological recordings revealed that modulating the electrophysiological properties of both POMC and cocaine- and amphetamine-regulated transcript (CART) neurons (POMC/CART neurons) and NPY/AgRP neurons are the possible mechanism of liraglutide-induced weight loss (Secher et al., 2014). Peripheral application of fluorescently labeled liraglutide binds GLP-1R within the ARC (Secher et al., 2014). Liraglutide depolarizes membrane potential and increases the spontaneous action potentials directly through postsynaptic GLP-1R in the ARC neurons expressing POMC (Secher et al., 2014; He et al., 2019). In peripheral pancreatic β cells, GLP-1 depolarizes membrane potential through activation of Na^+^-permeable TRPM4 and TRPM5 channels (Shigeto et al., 2015). Similarly, TRPC5 channels are involved in liraglutide-induced postsynaptic excitation of arcuate neurons (He et al., 2019). In addition to perikarya and dendrites expression, high level of GLP-1R was also observed in axons of ARC neurons (Farkas et al., 2021). Consistently, electrophysiological recordings showed that liraglutide increases the EPSCs frequency of POMC neurons suggesting the modulation of presynaptic excitatory synaptic transmission (He et al., 2019).

GABA released by the NPY/AgRP neurons is very important to the control of food intake probably *via* inhibiting the anorectic effects of the POMC neurons. Further electrophysiological study showed that, opposite to the effects on arcuate POMC neurons, GLP-1 hyperpolarizes arcuate NPY neurons indirectly *via* increased GABA_A_ receptor-mediated neurotransmission of local GABAergic interneurons (Secher et al., 2014; He et al., 2019). The Kisspeptin (Kiss1)-expressing neurons located in the ARC are responsible for gonadotropin-releasing hormone (GnRH)/luteinizing hormone (LH) release (Li et al., 2009; Han et al., 2015). The Kiss1 neurons may be a key integrator of metabolic status with GnRH/LH release. Liraglutide increases the action potential firing and causes a direct membrane depolarization of ARC Kiss1 cells in brain slices (Heppner et al., 2017).

Morphological studies demonstrated a particularly high density of GLP-1R expression in the PVN of mice (Cork et al., 2015), rats (Merchenthaler et al., 1999; Farkas et al., 2021), and primates (Heppner et al., 2015). Early study showed that exendin-4 induces diverse responses including depolarization, hyperpolarization, and no response in paraventricular hypothalamic neurons. The GLP-1-induced hyperpolarization of PVN neurons may be induced by an enhancement of inhibitory postsynaptic transmission (Acuna-Goycolea and van den Pol, 2004). Consistent with exendin-4-induced depolarization, Cork et al. (2015) also revealed that bath application of GLP-1 induces an inward current which is accompanied by an increase in membrane conductance. Activation of GLP-1R with exendin-4 enhances the amplitude but not the frequency of AMPA receptor-mediated EPSCs in PVN corticotropin-releasing hormone (CRH) neurons and thus promotes the excitability of CRH neurons postsynaptically (Liu et al., 2017). Functional studies revealed that activation of GLP-1R in the PVN reduces food intake (Larsen et al., 1997; McMahon and Wellman, 1998). Consistently, postnatal depletion of GLP-1R in the PVN increases food intake and induces obesity (Liu et al., 2017).

Different neural circuits have been proposed to maintain energy homeostasis. Both central GLP-1 and orexin pathways play an important role in neural integration of satiation and food reward. GLP-1 projections from NTS to NAc and VTA promote satiation and reduce food reward, while orexinergic projection from lateral hypothalamus to NTS suppresses satiation and increases food reward (Dossat et al., 2011). Early study revealed a direct modulation of GLP-1R on the electrophysiological activities of orexinergic neurons in the lateral hypothalamus. Application of exendin-4 depolarizes the membrane potential and increases the spontaneous discharge rate of orexinergic neurons in the lateral hypothalamus (Acuna-Goycolea and van den Pol, 2004). The GLP-1-induced excitation of orexinergic neurons is a directly postsynaptic effect that may be mediated by sodium-dependent non-specific cationic conductances. In addition, activation of GLP-1R enhances both glutamatergic and GABAergic neurotransmission presynaptically in orexinergic neurons. However, exendin-4 does not change the membrane potential as well as the firing rate of melanin-concentrating hormone (MCH) neurons in the lateral hypothalamus (Acuna-Goycolea and van den Pol, 2004). The GLP-1R activation-induced both postsynaptic and presynaptic modulation of orexinergic neurons may suggest some complex integration of satiation and food reward.

The paraventricular thalamic nucleus (PVT) neurons receive GLP-1 innervation from NTS and express GLP-1R (Cork et al., 2015; Farkas et al., 2021). PVT is involved in energy balance and reward control. Behavioral tests showed that intra-PVT application of exendin-4 reduces food intake and decreases food-seeking and food-motivated behaviors (Ong et al., 2017). Further electrophysiological recordings revealed that exendin-4 inhibits the spontaneous action potential firing in PVN neurons projecting to NAc core. Suppression of glutamatergic synaptic transmission may be associated with the reduced excitability of GLP-1R activation (Ong et al., 2017).

## Telencephalon

Moderate density of GLP-1R is expressed in both the cell bodies and fibers of the NAc shell and core (Cork et al., 2015; Heppner et al., 2015; Farkas et al., 2021). Activation of GLP-1R in NAc core induces suppression of food intake (Dossat et al., 2011; Mietlicki-Baase et al., 2014). Current-clamp recordings illustrated that exendin-4 induces a small reduction in evoked action potential from medium spiny neurons (MSNs) suggesting slightly postsynaptic effects. In addition to perikarya expression, GLP-1R is also expressed on the processes of NAc (Farkas et al., 2021) suggesting some possibly presynaptic modulation of the NAc activity. Indeed, further electrophysiological studies demonstrated that exendin-4 predominantly activates presynaptic GLP-1R in NAc to increase the frequency of AMPA/kainate receptor-mediated mEPSCs. Therefore, the enhancement of glutamatergic AMPA/Kainate signaling is probably involved in GLP-1-induced inhibition of food intake (Mietlicki-Baase et al., 2014). In addition to modulating food intake, recent publication revealed that NAc is also a possible molecular target for GLP-1-induced addiction behaviors (Hernandez and Schmidt, 2019; Hernandez et al., 2019). Intra-NAc application of exendin-4 increases the spontaneous firing rate of MSNs in cocaine-experienced rats and reduces cocaine-seeking behavior in rats (Hernandez et al., 2019).

Morphological studies revealed that the neurons in the bed nucleus of the stria terminalis (BNST) express a high level of GLP-1R (Cork et al., 2015; Heppner et al., 2015; Farkas et al., 2021). Application of GLP-1 elicits an inward current and depolarization accompanied by an increase in membrane conductance (Cork et al., 2015). Recently, under the model of cell-attached patch-clamp recordings, Williams et al. (2018) reported that GLP-1 induces either an increase or a decrease of spontaneous firing rate in GLP-1R expressing BNST neurons. Further whole-cell patch-clamp recordings revealed that GLP-1 induces either a depolarizing or hyperpolarizing response, while dopamine evokes response in a reciprocal fashion to that of GLP-1. The GLP-1-induced hyperpolarization is accompanied by an increase in membrane conductance suggesting the opening of potassium channels (Williams et al., 2018). In addition, functional study demonstrated that local injection of GLP-1 into the BNST induces food suppression during the dark phase (Williams et al., 2018).

Inconsistent distribution patterns of GLP-1R in the hippocampus have been reported by different morphological studies (Cork et al., 2015; Jensen et al., 2018; Farkas et al., 2021). For example, a relatively high level of GLP-1R-immunoreactivity was observed in mouse hippocampus (Jensen et al., 2018) while a low level of GLP-1R-immunoreactivity was revealed in rat hippocampus (Farkas et al., 2021), which may suggest some species difference of the GLP-1R expression in the hippocampus. However, functional studies did detect the effects of GLP-1R in the hippocampus. Early *in vivo* electrophysiological recordings showed that juxtacellular application of the active fragment of GLP-1, GLP-1 (7–36) amide induces an increase and then a decrease of firing activity in the hippocampal CA1 neurons. Modulation of non-NMDA glutamate receptor-mediated synaptic transmission is involved in GLP-1-induced effects (Oka et al., 1999). Bath application of GLP-1 induces a depolarization in most hippocampal neurons and a hyperpolarization in a few neurons (Cork et al., 2015). In addition, *in vitro* electrophysiological recordings further demonstrated that exendin-4 elicits an early fast excitatory response dose-dependently (Gullo et al., 2017). Consistent with the electrophysiological recordings, behavioral studies showed that activation of GLP-1R in the ventral hippocampal CA1 regions reduces food intake and body weight, while targeted ventral CA1 GLP-1R knockdown increases food-motivated behaviors (Hsu et al., 2015, 2018). In addition to modulating feeding behaviors, GLP-1 promotes the proliferation of progenitor cells and increases immature neurons in the hippocampus and in turn reverses memory impairment (Lennox et al., 2014). Activation of GLP-1R with liraglutide improves cognition decline of db/db mice *via* increasing neuronal survival in the CA1, CA3, and DG regions of hippocampus (Zhang et al., 2021).

The olfactory bulb is the basic brain region responsible for olfactory information. The deep short axon cells (dSACs) in the granule cell layer (GCL) of olfactory bulb, named PPG neurons, could synthesize and release GLP-1 and in turn modulate the activity of the first-order neurons, mitral cells (MCs) which are the primary projection neurons of the olfactory bulb (Thiebaud et al., 2016). Positive expression of GLP-1R is detected in the GCL of olfactory bulb (Cork et al., 2015). Patch-clamp recordings revealed that bath application of GLP-1 or exendin-4 increases the spontaneous firing frequency and decreases the excitation threshold for MC firing in olfactory bulb. Decreasing the conductance of voltage-dependent potassium channels, Kv1.3, is the possible ionic mechanism of GLP-1-induced enhancement of MC excitability (Thiebaud et al., 2016). Recently, further studies revealed that optogenetic activation of PPG neurons in the GCL generates biphasic inhibition-excitation response in MCs. However, a single pulse light stimulation of PPG neurons produces only glutamatergic EPSCs, but not IPSCs, in granule cells. The stimulation of PPG neurons-induced glutamatergic EPSCs is much faster than that of GABAergic IPSCs in MCs. Under the condition of blocking GABAergic neurotransmission, light stimulation of PPG neurons results in an increase in the excitation of MCs suggesting the involvement of PPG neurons in shaping the MC firing patterns (Thiebaud et al., 2019). It is known that, in addition to olfactory physiology, MC activity is also associated with feeding and nutritional status (Fadool et al., 2011; Aimé et al., 2014; Thiebaud et al., 2014; Riera et al., 2017). The olfactory acuity is regulated by the metabolic state and therefore the olfactory system is a driver of feeding behavior. Enhancement of neuronal excitability of the major output neurons of the olfactory bulb *via* blocking voltage-dependent potassium channel reduces body weight in obese mice (Schwartz et al., 2021). Previous study suggested that chronic administration of fat in the diet impairs the spontaneous firing rate of MCs (Fadool et al., 2011), and reduces the amplitude of electro-olfactogram (EOG). Furthermore, the volume of olfactory bulb is significantly smaller in individuals with obesity and negatively correlated with body mass index (BMI) (Poessel et al., 2020). Therefore, the GLP-1-induced excitation of MCs, probably *via* inhibition of voltage-dependent potassium channel conductance and enhancement of glutamatergic neurotransmission, could lead to changed excitability of higher olfactory cortical as well as hypothalamic regions to change metabolic states.

## Conclusion

Being a peptide involved in the regulation of food intake and energy metabolism, GLP-1 has been demonstrated to suppress food intake and reduce body weight. In this review, we provide a description of recent advances of GLP-1-induced inhibition of feeding behaviors and modulation of neuronal electrophysiological activities in multiple brain nuclei located within the medulla oblongata, pons, mesencephalon, diencephalon, and telencephalon (Table 1). Activation of GLP-1R suppresses food intake and induces postsynaptic depolarization of membrane potential (Figure 1A) and/or presynaptic modulation of glutamatergic or GABAergic neurotransmission (Figure 1B). Several ionic mechanisms such as non-selective cation channel, voltage-dependent potassium channel, and TRPC5 channel may be associated with activation of GLP-1R-induced electrophysiological effects (Figure 1A). This review may provide a rationale about the cellular mechanisms of GLP-1-induced suppression of feeding behaviors.

## Author Contributions

X-YC wrote the original draft. LC revised the manuscript. WY and A-MX contributed to the conception, design, and revision of the manuscript. All authors contributed to the article and approved the submitted version.

## Conflict of Interest

The authors declare that the research was conducted in the absence of any commercial or financial relationships that could be construed as a potential conflict of interest.

## Publisher’s Note

All claims expressed in this article are solely those of the authors and do not necessarily represent those of their affiliated organizations, or those of the publisher, the editors and the reviewers. Any product that may be evaluated in this article, or claim that may be made by its manufacturer, is not guaranteed or endorsed by the publisher.
